# Trial protocol: a parallel group, individually randomized clinical trial to evaluate the effect of a mobile phone application to improve sexual health among youth in Stockholm County

**DOI:** 10.1186/s12889-018-5110-9

**Published:** 2018-02-05

**Authors:** Anna Nielsen, Ayesha De Costa, Aspasia Bågenholm, Kristina Gemzell Danielsson, Gaetano Marrone, Jens Boman, Mariano Salazar, Vinod Diwan

**Affiliations:** 1 Stockholm County Council. Department of Women’s and Childrens Health K6, Karolinska University Hospital Solna, 17176 Stockholm, Sweden; 2 Department of Public Health Sciences, Karolinska Institutet, Tomtebodavägen 18 A, 171 77 Stockholm, Sweden; 3 Department of Women’s and Children’s Health K6’, Karolinska University Hospital Solna, 17176 Stockholm, Sweden; 4 Department of Clinical Microbiology, Umeå Universitet, SE-90185 Umeå, Sweden

**Keywords:** Pragmatic randomized controlled trial, Youth, *Chlamydia trachomatis*, mHealth, smart-phone application

## Abstract

**Background:**

Genital *Chlamydia trachomatis* infection is a major public health problem worldwide affecting mostly youth. Sweden introduced an opportunistic screening approach in 1982 accompanied by treatment, partner notification and case reporting. After an initial decline in infection rate till the mid-90s, the number of reported cases has increased over the last two decades and has now stabilized at a high level of 37,000 reported cases in Sweden per year (85% of cases in youth). Sexual risk-taking among youth is also reported to have significantly increased over the last 20 years. Mobile health (mHealth) interventions could be particularly suitable for youth and sexual health promotion as the intervention is delivered in a familiar and discrete way to a tech savvy at-risk population. This paper presents a protocol for a randomized trial to study the effect of an interactive mHealth application (app) on condom use among the youth of Stockholm.

**Methods:**

446 youth resident in Stockholm, will be recruited in this two arm parallel group individually randomized trial. Recruitment will be from Youth Health Clinics or via the trial website. Participants will be randomized to receive either the intervention (which comprises an interactive app on safe sexual health that will be installed on their smart phones) or a control group (standard of care). Youth will be followed up for 6 months, with questionnaire responses submitted periodically via the app. Self-reported condom use over 6 months will be the primary outcome. Secondary outcomes will include presence of an infection, Chlamydia tests during the study period and proxy markers of safe sex. Analysis is by intention to treat.

**Discussion:**

This trial exploits the high mobile phone usage among youth to provide a phone app intervention in the area of sexual health. If successful, the results will have implications for health service delivery and health promotion among the youth. From a methodological perspective, this trial is expected to provide information on the strength and challenges of implementing a partially app (internet) based trial in this context.

**Trial registration:**

ISRCTN 13212899, date of registration June 22, 2017.

## Background

*Chlamydia trachomatis (C.trachomatis)* and other sexually transmitted infections (STIs) are considered a major public health problem worldwide affecting mostly youth [1]. The World Health Organisation estimates that 357 million new infections with one of four STIs: Chlamydia, gonorrhoea, syphilis and trichomoniasis occur each year [1]. *C.trachomatis* is the most commonly reported STI worldwide. If untreated, *C.trachomatis,* as well as some other bacterial infections, could lead to adverse reproductive health outcomes such as infertility [2]. *C.trachomatis* also increases susceptibility to HIV infection [3]. In addition, drug resistance, is a major threat to reducing the impact of STIs worldwide [1].

An objective comparison of *C.trachomatis* prevalence between countries is difficult as reporting procedures and national screening practices vary widely [4]. There are large differences in *C.trachomatis* control policies across Europe and worldwide. Sweden, Denmark, UK are all examples of countries with well-functioning screening programs [5].

### C.Trachomatis in Sweden

Sweden has a long tradition of testing. An opportunistic screening approach was introduced already in 1982 offering *C.trachomatis* testing to women requesting contraceptives, antenatal care or abortion, male partners of infected women were also tested (contact tracing) [6, 7]. Then, in the late 80s the infectious disease law was changed and came to include free testing and treatment of *C.trachomatis*, partner notification and case reporting [8]. The incidence of *C.trachomatis* and its sequelae decreased up until the mid-1990s [6, 7, 9]. The Swedish screening program was held up as an example of success in bringing down *C.trachomatis* infection rates [10].

Despite this initial positive effect of widespread testing, and despite significant efforts, including increasing availability of testing and treatment services, the incidence of *C.trachomatis* in Sweden has, over the past two decades, steadily increased and has now stabilized at a high level of approximately 37,000 reported cases per year. Youth aged 15–29 constitute the majority (85%) of all cases [11].

The high rates of *C.trachomatis* infection, notwithstanding the resources and efforts invested, caught the attention of several stakeholders in Sweden. A National Plan for Chlamydia Prevention was launched in 2009. This resulted in initiatives to increase condom use, improve knowledge about *C.trachomatis* infection and the consequences of unprotected sex, i.e. focusing on primary prevention strategies, but also increase the availability of testing and treatment services for infection i.e. secondary prevention strategies [12].

### Sexual behavior of youth in Sweden

As a response to the increasing level of *C.trachomatis* in the country, young people’s sexual habits have been surveyed in studies in Sweden [13–15]. Sexual risk-taking among youth has significantly increased over the last 20 years, especially among young women [16]. Although knowledge and confidence in condoms as an effective way of prevention of STI has increased over the last decades, this has not resulted in more widespread use [17]. In a large national population based study, 50% of women and 40% of men between the ages of 18 and 30 stated that they never or seldom used condoms with a temporary partner [14]. In a national survey with 15,000 youth aged 15–29, about 70% reported not using a condom at the last intercourse and 50% said they did not use a condom with a new partner at the last intercourse [13].

Many studies have focused on finding and evaluating how to best tailor effective sexual health interventions [18–20]. The effects of interventions vary. Educational interventions show positive results in terms of increased knowledge regarding STI and condom use, and even in attitude changes [21]. However, increased knowledge about STI does not necessarily translate into a positive impact on behavior (increased condom use, reduction in sexual partners) [22]. A motivational behavioural intervention was evaluated in a large randomised study in US, but the intervention did not have any effect on condom use [23]. Motivational Interview (MI) as a method to change sexual risk behaviour has shown mixed effectiveness in Sweden [24, 25]. With increasing demands of availability and increasing number of *C.trachomatis* tests being performed, it can be argued that scaling up of successful MI techniques and other forms of face-to-face counseling are time consuming, and may not be feasible for already time-constrained staff to implement. Thus, there is an urgent need for new innovative ideas to promote safe sex among young people.

### mHealth interventions to promote safe sexual practices

Mobile health – mHealth is an increasingly popular way to deliver health promotive information to large parts of the public at low cost and serve as a complement to ongoing activities within the health care system. mHealth interventions could be particularly suitable for youth and sexual health promotion as the intervention is delivered in a familiar and discrete way to a tech savvy at-risk population [26]. mHealth also offers the possibility of a range of different interventions for example reminder (to use a condom or to test), and motivational messages, peer feedback – each of which allows differing levels of engagement [27]. Furthermore creative mHealth interventions such as games, videos, and challenges (quizzes, puzzles etc) could engage youth in sexual health awareness and behavioral change readiness. The coverage of mobile phone ownership among young people in Stockholm County is high. Among the population in Sweden 97% own a mobile phone and the majority of these are smart phones [28].

A systematic review of randomized controlled trials (RCTs) of sexual health interventions delivered by mobile technologies was recently published [27]. While there is promising evidence of the impact of mHealth on increased knowledge about STI, and increased levels of testing for STI, there is still a need for better developed interventions that are more engaging and aimed at behavioral changes i.e. increased condom use. Given the high use of mobile phones among the youth of Stockholm, and the high rates of *C.trachomatis* in this group, a well-designed mHealth intervention merits testing here. Furthermore, there has been no previous evidence from Sweden on the use of mHealth interventions to promote safe sexual practices among the youth. If the interventions can increase condom use and decrease the occurrence of *C.trachomatis* this will have implications in terms of supporting the opportunistic screening program by complementing its preventive arm. This could reduce costs for the healthcare system; testing and treatment are costly, more so, there are some indications of antibiotic use lead to resistance among *C.trachomatis* [29]. Such an intervention could be potentially delivered to youth across wide geographic areas at low cost.

### Objective

The aim of this pragmatic randomized trial is to compare the effectiveness of a mobile phone application to improve sexual health among youth of Stockholm County to routine care. Safe sexual practices will include self-reported condom use (primary) and *C.trachomatis* positivity (secondary).

## Methods

**Fig. 1:**
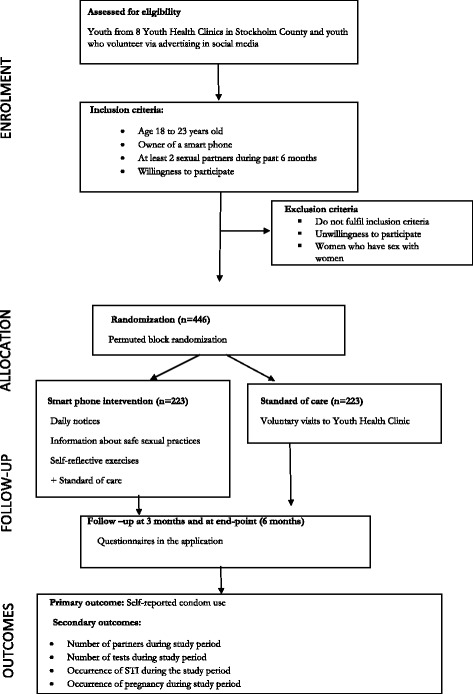
Flow diagram

This study is a multicentre (8 recruiting clinics), parallel group randomised controlled trial. Eligible participants are randomised in a 1:1 allocation ratio to one of two arms: an intervention arm, in which youth receive the interactive application (app) called the ‘Skyddslaget’ or ‘protection team’ app in addition to the standard of care (defined below); and a control arm in which they receive only the standard of care Fig. 1.

### Study setting

The settings for this trial are the Youth Health Clinics (YHC) in Stockholm County. These youth clinics are part of nationwide network of such clinics in Sweden. The target age of clients attending the YHC ranges from 12 to 23 years old. Besides contraceptive counselling services and the provision of contraceptives to youth, the clinics are involved both in primary prevention of STI, i.e. sexual education to school classes, and secondary prevention i.e. testing, contact tracing and treatment services. Condoms are distributed for free at the YHC. The YHCs therefore provide a ‘one stop shop’ service with regard to sexual health for young people. Each clinic is staffed by nurses/midwives, behavioural therapists, social workers and a physician. In the whole country there are approximately 250 clinics, and in Stockholm County there are 33 YHC. The trial will be conducted at 8 of 33 YHC in Stockholm County.

### Inclusion and exclusion criteria

All youth over 18 years old who visit any of the eight YHCs in Stockholm, as well as all youth between the ages of 18–23 years old who register interest to participate via the trial website, will be potential participants for the trial if they also meet other inclusion criteria. Youth included in the trial should (a) own a smart phone and (b) should have had at least two sexual partners during the previous 6 months. This second criteria is to ensure youth included in the trial have recently had sex outside a stable relationship. Youth who fulfil the inclusion criteria but do not wish to participate will be excluded from the trial. Women who have sex with women will be excluded from the trial as the intervention focuses on condom use.

### Recruitment

Youth who attend any of the eight YHC and who fulfil the inclusion criteria will be informed about the study and asked about willingness to participate. In order to facilitate the recruitment process and identify eligible participants, midwives at each of the study sites will be carefully informed about the study. At time of the recruitment a member of the research team or the midwife will provide information to youth who show interest in participating in the trial.

Youth can also register interest in participating in the trial via the trial website (www.skyddslaget.se). These potential participants will be contacted by the research team. Those who fulfil the inclusion criteria will meet a member of the research team who will provide information about the trial.

### Informed consent

All eligible youth who express an interest will individually be given an initial verbal description of the proposed study by a trained member of staff. They will then be presented a written informed consent form in Swedish.

### Randomization

Stratified randomization, separately for male and female participants, will be performed. Within each stratum, block randomization will be used to ensure balanced representation in the two treatment arms (control and intervention) as recruitment progresses. Blocks of 4 and 6 will be used. Randomisation will be done electronically by an interactive web response system at the time of recruitment. Once participants have consented, each participant will be given a study identification number at the site and instructed in the protocols based on their randomisation group. This will allow allocation concealment at the sites.

### Intervention

The intervention which will be evaluated in this trial will consist of “Skyddslaget” app, a mobile phone app to promote safe sex among the youth. This intervention will be compared against standard of care.

### Description of intervention

This smart phone application was developed based on individual interviews, focus group discussions with youth in Stockholm, and considering health behavior change principles.

The intervention will be in the form of a smart phone application, the “Skyddslaget” app, which will be installed onto smartphones of those applicants allocated to the intervention arm of the trial. The app is intended to deliver ‘safe-sex and STI’ relevant snippets of information (facts) to participants on their phones in a youth friendly format. In addition it will also have a more interactive and engaging element which will include weekly challenges (games) and quiz, as well as personal stories related to safe sex narrated by peers. Activities/information snippets within the app will be dynamic, i.e. change periodically over time. The intervention will run over a period of 6 months from enrolment into the study.

### Description of the control

The participants in the control arm will also receive an app, which will be a dummy application that will be downloaded onto their phones. This app will not provide an information or have any interactive modules but will only contain only the study baseline and follow-up questionnaires.

### Standard of care

Both the control and intervention groups will receive the standard of care which is as follows:

Guided by national laws and regulations, regional policies control the preventive work in the different County Councils in Sweden [30]. Standard of care for youth visiting the YHCs in Stockholm County includes exploring sexual behaviour of the individual, give recommendations and prescription of contraceptives, information about and distribution of condoms, testing for STI upon request from the youth and/or upon recommendation from the staff. If the STI test is positive the youth is called back to the clinic for treatment and contact tracing. A test of cure and/or re-testing after a positive test is not routinely recommended. The standard operating procedures (SOP) might differ between different YHCs.

Participants recruited outside the clinics (via the trial website) will be receive the standard of care as above should they choose to visit the YHC.

### Outcome measures

### Primary outcome

Self-reported condom use during the past 6 months.

### Secondary outcomes

Number of partners during the study period. Number of tests for *C. trachomatis* during the study period. Occurrence of pregnancy during study period occurrence of STI during the study period.

### Outcome assessment

Primary outcome: self-reported condom use (number of sexual partners when condom was always used/total number of sexual partners in percentage) during the past 6 months. This 6 months measure will be computed by summing of data provided at 3 months and 6 months after enrollment. (Each measure will refer to the previous 3 months).

Secondary outcomes: Self-reported number of partners (mean); number of STI test (mean); occurrence of STI (yes/no); and occurrence of pregnancy (yes/no) during the study period will be assessed at 6 months.

For self-reported number of partners, the mean number of partners per 3 month period will be calculated. This will be based on the average of data provided at the 3 months and 6 months assessment questionnaires. (Each measure will refer to the previous 3 months). For the other two secondary outcomes, a ‘yes’ response at any point in the study will be considered.

### Duration of study

The end of study is defined as day 180 after the day of recruitment (day 1). Trial participants will provide data on outcome measures at baseline, and at 3 months and 6 months following recruitment. All data will be submitted via the app.

### Study procedures: Data collection and follow ups

There are no in person follow ups intended in this study, though the patient will be informed that s/he can contact the research team/study clinic at any point during the study. Data will be collected at three time points – at baseline, at month 3 and month 6. All data will be sent in by participants via the app. Appropriate security measures to ensure safe and confidential transfer of data onto a safe haven server have been made.

The trial team will make efforts to ensure that participants respond to the data requests at month 3 and 6. Youth often lead a life full of variety in terms of living arrangements and relationship status. In addition, smart phones might break, be lost, and phone numbers might be changed. Therefore, obtaining data at the required time points could prove a challenge. Participants will consent to a research assistant contacting them, if responses via the app are not received. Three attempts at contact will be made within 10 days of the expected date of receipt. If the participant is not traced/does not respond during this time, he/she will be considered lost to follow up.

### Quality control

SOP for key processes in the study have been developed. Prior to recruitment, the site team will receive extensive training on the objectives, methods and processes of the study. Quality assurance protocols will be set in place to maintain quality of processes in the clinics.

### Database

Data submitted via the app by the participant will be electronically routed onto a database that will be stored on a secure server. At the end of the study this database will be locked prior to analysis.

### Sample size

Sample size has been estimated on the assumption that the smart phone intervention to promote safe sexual practices is expected to increase the self-reported condom use among sexually active youth in Stockholm County by 20% i.e. from 50% to 70%.

The minimum sample size was estimated to be equal to 268, half (*n* = 134) allocated to receive the smart phone application and the other half (*n* = 134) to receive the control. This sample size is sufficient to detect the estimated difference in self-reported condom use between the two groups with 90% power at significant level α = 0, 05. Loss to follow up in similar internet based trials has been reported at a level of 20–40% [31, 32]. Loss to follow up in the present study was calculated at 40% in order to be more conservative. This would imply a final sample size of 446 i.e. 223 in each arm.

### Statistical analysis

Analysis will be based on an intention-to-treat approach. Data will be summarized with descriptive statistics (mean, median, standard deviation, percentiles for numerical variables, frequencies and percentages for categorical variables). Cross tabulations with Chi-Square or Fisher test will be used to test for un-adjusted relationship between binary outcomes and categorical independent variables. For numerical variables t-test, Wilcoxon rank sum test and Kruskal Wallis test will be used to compare mean and medians in two and more groups respectively. A binary logistic regression model will be used to identify significant predictors of binary outcomes. Crude and adjusted odds ratios (OR) with their 95% confidence intervals will be presented. Ordinal or linear regression models will be used to identify significant predictors of numerical outcomes. *P*-value < 0.05 will be considered significant in the final models. Missing values will be filled in (imputed) with a “missing at random” assumption. Sensitivity analysis will be made to explore the effects of departures from the assumptions made in the main analysis. Sub-group analysis, depending on the way on recruitment (clinic or website), and sex will be made.

### Ethical aspects

The study protocol has been ethically approved by institutional ethical review board of the Stockholm region (reference number: 2017/651–31/4). The rights and welfare of the participants will be protected according to the Declaration of Helsinki Ethical Principles for Medical Research Involving Human Subjects. Clinical care and emergency medical services are provided by the participating institutions. Data that is collected as part of the study will be not be linked to any individual, personal identifiers will not be used in data storage, and confidentiality will be maintained at all levels of data management.

Exploring people’s sexual habits and condom use is sensitive information and requires reliable measures to ensure data privacy protection. The Swedish Personal Data Act, 1998:204 is and will be followed throughout the trial. Only members of the research team have access to data collected in the project. Data files are stored on secure servers accessible only on pass worded computers used solely by the researchers. To secure the data transfer from the app, i.e. surveys filled in by participants, all data was encrypted by using a Secure Sockets Layer (SSL) when it was transferred from the app to the server.

### Other studies

We aim to conduct a qualitative study just after the end of the trial. The focus of the qualitative evaluation will be to look deeper into the experience of receiving the smart phone application; what parts of the application might have been perceived as being helpful and what parts might have been intimidating, and why the youth perceived that it did or did not influence their behaviour. We plan to do individual interviews with a sample of youth from the intervention arm and hold focus-group discussions with 6–10 participants in each group.

## Discussion

The popularity of mobile health apps is evident from the estimated 102 billion downloads of health-related apps worldwide [33]. Swedish governmental health strategy strongly supports digitalisation of the health service with a focus on self-managed and remote care [34]. The stigmatised nature of STIs and youth they most affect (almost all young adults in Sweden own a smartphone and are very familiar with digital technology) could suggest that provision of information about STIs, testing and treatment through apps is an effective and highly feasible medium for those at highest risk.

A recent review [33] of current mobile apps for STIs (and genital infections) indicated that few apps meet the needs of people seeking comprehensive, accurate sexual health information and that there was a pressing need for high-quality, easily identifiable apps that address the most common STIs and health concerns. The app to be tested in this trial is developed by public health professionals and clinical staff working in the youth clinics, and it was developed with the input from young people in Stockholm County.

It will provide new information on the level of success with this modality with the youth and also provide information on the strengths and pitfalls of trials partially done over the net in this context.

There has also been recent interest in trials that can be completely or partially conducted over the internet [35]. It is possible to recruit participants, allocate interventions, measure outcomes and enter data using the internet. This trial will have combined traditional trial methods as well as digital app (internet) based trial methods. (See table 1.)

**Table 1:** Trial step.

Trial step	Traditional (face to face)	Over the app (internet)
Recruitment	√	√ (net)
Consent	√	
Randomisation		√
Baseline data collection		√
Follow up week 12		√
Follow up week 24		√
End of study communication		√

There are potential advantages and disadvantages of conducting part or all of a randomized trial over the internet. The internet potentially provides researchers with the ability to reach potential participants who would otherwise be unreachable, to access non-clinical populations and to reduce research costs [35]. The advantages of conducting an online trial include the potential to improve the trial’s external validity when an online intervention is being evaluated, and users have the ability to self-refer without having to participate via a health professional; the randomization process has the potential to be more secure, with allocation being concealed [36]. Despite these advantages, the authors indicate that online recruitment could result in unrepresentative samples and multiple registrations and increases the difficulty of verifying identity. They also point to the potential loss of power in the study if participants assigned to the intervention do not receive or fail to complete the intervention, resulting in the true effect of the intervention being underestimated. We are uncertain about the extent to which these problems will affect the current trial. We have tried to anticipate them though, and have put in place protocols for reminders in case of non-response, both of which will be quantified. Loss to follow-up is of particular concern in online trials. Although ours is a mixed trial, we have assumed a large loss to follow up which has been built into the sample size.

In conclusion, this trial attempts to exploit the high mobile phone usage among youth to provide a phone based app intervention in the area of STI, which disproportionately affects youth. If successful, the results will have implications for health service delivery and health promotion among the youth. From a methodological perspective, this trial is also expected to provide important information on the strength and challenges of implementing a partially app (net) based trial in this context.

## Abbreviations

App: Application
*C.trachomatis*: *Chlamydia trachomatis*
HIV: Human immunodeficiency virus
mHealth: Mobile health
MI: Motivational interviewing
SOP: Standard operating procedure
SSL: Secure sockets layer
STI: Sexually transmitted infection
YHC: Youth health clinic
